# Analysis of High‐Risk Factors for Failure of Bleeding Control in Enhanced Myometrial Vascularity Treated With Uterine Artery Embolization

**DOI:** 10.1002/jcu.70341

**Published:** 2026-07-14

**Authors:** Linhua Zhu, Yongfang Yue, Lili Cao

**Affiliations:** ^1^ Women's Hospital School of Medicine, Zhejiang University Hangzhou China; ^2^ Hangzhou Linping District Maternal & Child Health Care Hospital Hangzhou China

**Keywords:** EMV location, enhanced myometrial vascularity, uterine artery embolization, uterine EMV diameter

## Abstract

**Purpose:**

Enhanced myometrial vascularity (EMV) is a condition frequently linked to abnormal uterine bleeding during pregnancy, leading to significant morbidity, including severe bleeding and anemia. It poses a considerable economic burden on healthcare systems. This study aims to explore the factors influencing treatment outcomes in patients undergoing uterine artery embolization (UAE) for bleeding from EMV.

**Methods:**

A retrospective analysis was performed on 110 patients treated with UAE in the Women's Hospital, Zhejiang University School of Medicine, yielding a success rate of 94 out of 110 cases. Sixteen patients with treatment failure were included in the study group, and 94 successfully treated patients were included in the control group.

**Results:**

Key findings revealed that patients in the study group were younger, presented with larger uterine EMV diameters, had longer intervals since their last uterine surgery, and more frequently exhibited uterine fundal involvement (*p* < 0.05). Binary logistic regression analysis identified diameter of uterine EMV (OR = 3.23, 95% CI: 1.59–8.51, *p* < 0.05) and uterine fundal involvement (OR = 74.03, 95% CI: 7.88–697.40, *p* < 0.05) as significant risk factors for treatment failure. Postoperative follow‐up demonstrated that the treatment failure group had significantly prolonged bleeding times and a higher incidence of unchanged or enlarged malformations compared with the successful treatment group (*p* < 0.05).

**Conclusion:**

These findings suggest that EMV size and uterine fundal involvement are critical determinants of UAE treatment failure, providing important evidence for clinical risk assessment and future research on the management of EMV‐related bleeding.

## Graphical abstract

1. Enhanced Myometrial Vascularity (EMV) is a serious gynecological condition that often leads to abnormal uterine bleeding.
2. Malformation size and involvement of the uterine fundus are significant risk factors.
3. If EMV diameter > 3.85 cm, the bleeding of the patient is more likely to result in failure in UAE treatment.

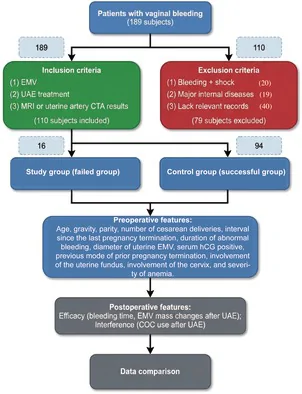

## 1 Introduction

Enhanced myometrial vascularity (EMV) is classified as either a uterine arteriovenous malformation or a uterine artery pseudoaneurysm. It has gained increasing clinical attention in recent years because of its strong association with abnormal uterine bleeding during pregnancy. This condition can result in significant discomfort and complications for patients, leading to higher healthcare costs and emotional distress (Algeri et al. 2023). Clinical management of EMV is individualized and consists of multiple strategies. Medical therapy, such as gonadotropin‐releasing hormone agonists (GnRH‐a), serves as a conservative option for stable patients with mild symptoms and low‐velocity flow, which can reduce uterine perfusion and promote lesion regression. As an effective alternative, laparoscopic surgery has also been applied. Laparoscopic compression suture of the hypervascular lesion combined with bilateral uterine artery ligation achieves reliable hemostasis with minimal trauma and fast recovery. Uterine artery embolization (UAE) is the most widely adopted minimally invasive intervention, providing rapid and effective devascularization to control bleeding while preserving uterine function and fertility (Le and Nguyen 2025). While the UAE shows a variable success rate, it has a risk of treatment failure, underscoring the need for further investigation. Understanding the mechanisms and factors contributing to EMV is essential for improving patient outcomes.

It is well known that transvaginal ultrasound is used for initial screening, and MRI is added for difficult cases; their combined application optimizes diagnostic accuracy and supports clinical decision‐making. Research found that for postmenopausal bleeding, endometrial thickness and uterine artery Doppler indices effectively predict endometrial cancer, with high sensitivity and specificity; endometrial thickness < 4 mm can safely rule out malignancy (Nguyen and Nguyen 2022). For uterine sarcoma, ultrasound shows typical features including a solitary mass, heterogeneous echo, invisible endometrium, cystic changes, and moderate‐rich vascularity, while expert consultation ultrasound markedly improves sensitivity. MRI serves as a valuable complementary modality, especially for atypical uterine masses and suspected sarcoma, offering high sensitivity in distinguishing malignant from benign lesions, assessing invasion depth, and preoperative staging (Nguyen et al. 2025). Also, gynecologic ultrasound is the first‐line screening method for the EMV. It demonstrates characteristic anechoic areas, mosaic turbulent flow, and high‐velocity low‐resistance spectra, offering noninvasive, convenient, and repeatable evaluation (Xholli et al. 2024). Furthermore, characteristic vascular patterns demonstrated on ultrasonography also contribute to the differential diagnosis of EMV (Nguyen and Nguyen 2023). MRI serves as an important complementary tool. Combined use improves diagnostic accuracy and provides key evidence for treatment planning (Tsang and Gomez 2026).

Previous studies have demonstrated that various phenotypic factors, such as patient age and EMV diameter, may influence outcomes following UAE. Correlations between these factors and EMV severity have been established, suggesting the potential for tailored therapeutic approaches to optimize patient management (Xholli et al. 2022). Examining the clinical features of EMV is therefore important for elucidating its impact on patient care and for guiding the development of more effective treatment strategies.

The present study employed a retrospective design using comprehensive clinical data from patients diagnosed with EMV who underwent UAE. This approach enables the evaluation of real‐world outcomes over an extended period and provides a robust framework for identifying risk factors associated with treatment failure. The specific objectives were to describe the clinical characteristics of EMV patients and to assess predictors of UAE treatment outcomes.

## 2 Materials and Methods

### 2.1 Subjects

This retrospective study included patients diagnosed with EMV who attended the Department of Obstetrics and Gynecology between January 2013 and January 2023. The study was approved by the Ethics Committee of Women's Hospital, Zhejiang University School of Medicine (Approval No.: IRB‐20250059‐R). Informed consent was waived by the Ethics Committee due to the retrospective nature of the study, in accordance with local regulations. A schematic flow chart of data collection is shown in Figure 1.

**FIGURE 1:**
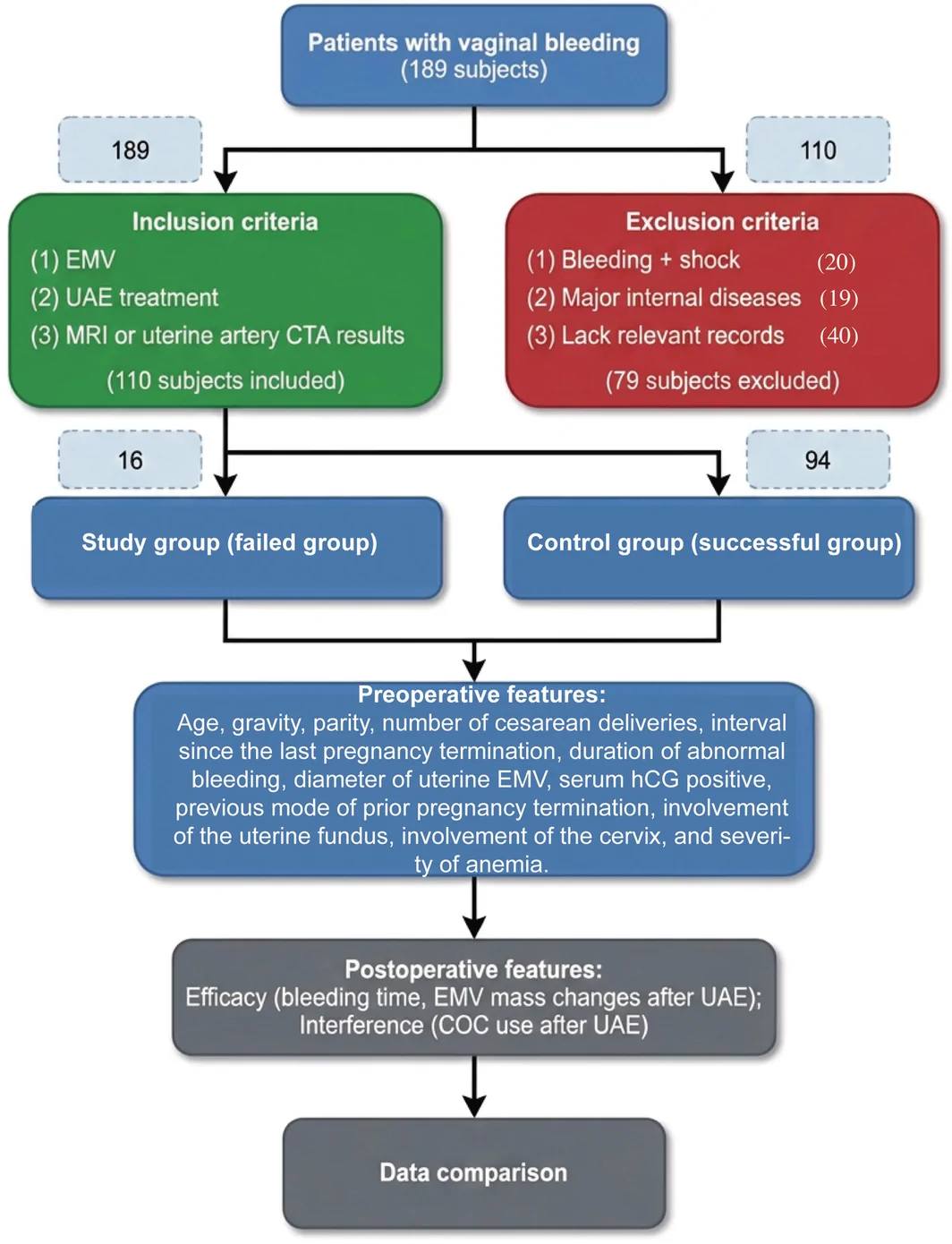
The study flowchat.

Patients were eligible for enrollment if they met the following requirements: a diagnosis of pregnancy‐related EMV accompanied by abnormal uterine bleeding, treatment with UAE, and availability of diagnostic imaging consisting of either pelvic magnetic resonance imaging (MRI) or uterine artery computed tomography angiography (CTA) (Narang et al. 2015). Exclusion criteria were defined as follows: patients presenting with hemorrhage complicated by shock, those with severe medical conditions including cardiac disease or coagulation disorders, and individuals with incomplete or missing medical records that precluded full data analysis.

Typical images of EMV obtained by ultrasonography, MRI, and CTA are presented in Figure 2.

**FIGURE 2:**
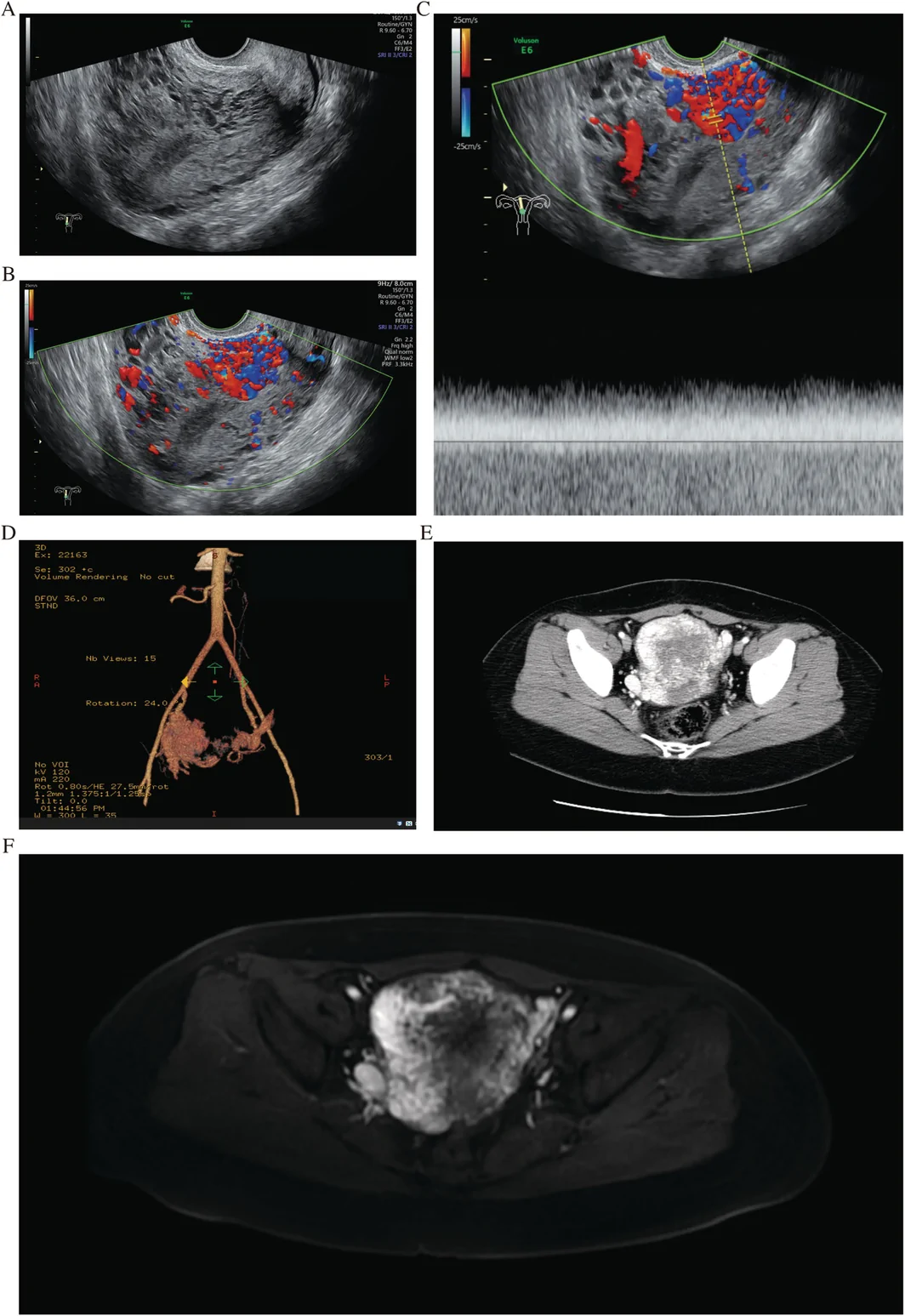
Typical images of EMV obtained by ultrasonography, MRI, and CTA. (A–C) images obtained by ultrasonography. (D, E) images obtained by MRI. (F) images obtained by CTA.

Successful treatment was defined as cessation of vaginal bleeding without the need for further intervention within 3 months after UAE. Patients meeting this criterion were included in the control group, while those with treatment failure were assigned to the study group.

### 2.2 Treatment Methods

In the vast majority of clinical cases, operators place a 5‐F introducer sheath manufactured by Medikit (Tokyo, Japan) via a single‐side femoral artery access. Under fluoroscopic guidance, a 5‐F pigtail catheter (Fansac IV, Terumo‐Clinical Supply, Gifu, Japan) is delivered into the abdominal aorta, which serves as the preliminary step before superselective cannulation of the uterine artery (UA). Diagnostic pelvic aortography is subsequently conducted to delineate the vascular architecture of pelvic arteries, with meticulous assessment focused on accessory blood vessels aside from the main UA.

Selective intubation of bilateral uterine arteries is completed with the assistance of a 5‐F cobra catheter paired with a 0.035‐in. angled Radifocus guidewire (Terumo, Tokyo, Japan), alongside a conventional coaxial 2.8‐F Renegade microcatheter from Boston Scientific Corp. Vascular occlusion is then accomplished via injection of gelatin sponge particles (560–710 μm, Hangzhou Alikang Company) using the manual pumping technique. Once bilateral UAE is finished, obstetric specialists conduct bedside physical assessment to verify satisfactory clinical hemostasis. In instances where ongoing active hemorrhage cannot be controlled, alternative aberrant feeding vessels are identified and subjected to supplementary embolization.

### 2.3 Study Parameters

Clinical features recorded before surgery included age, gravity, parity, number of cesarean deliveries, interval since the last pregnancy termination (the duration from the last pregnancy event to the identification of EMV), duration of abnormal bleeding, diameter of uterine EMV (To ensure the comparability of measurements, the diameter of uterine EMV was defined as the longest diameter of EMV measured by preoperative ultrasonography), serum hCG positive, previous mode of prior pregnancy termination, involvement of the uterine fundus, involvement of the cervix, and severity of anemia.

Postoperative data collected from both groups included two efficacy indicators (bleeding duration after UAE and EMV mass changes after UAE) and one confounding factor (use of compound short‐acting oral contraceptives [COCs] after UAE). Additionally, transvaginal ultrasound was performed at one and 3 months to assess EMV mass changes following UAE.

### 2.4 Statistical Analysis

Statistical analyses were conducted using SPSS version 23.0 (IBM Corp., Armonk, NY, USA) and R version 4.3.1 (R Foundation for Statistical Computing, Vienna, Austria). The Shapiro–Wilk (S‐W) test was adopted to verify the normal distribution of data. For data with a normal distribution and homogeneity of variance, results were expressed as mean ± standard deviation, and the *t*‐test was applied. For data not meeting these assumptions, a nonparametric test was used, and results were expressed as median (25th–75th percentile). The chi‐square test (*χ* ^2^ test) was employed for categorical variables, with results presented as case numbers and percentages. Fisher's exact test was used for categorical comparisons with expected cell counts ≤ 5 to avoid inaccuracies from the chi‐square test. Firth‐corrected binary logistic regression was performed to evaluate the influence of age, parity, time since the last uterine surgery, diameter of uterine EMV, involvement of the uterine fundus, and involvement of the cervix on the failure of UAE (comparison of baseline data: *p* < 0.05). A *p* < 0.05 was considered statistically significant.

The positive predictive value and negative predictive value of risk factors were determined using the ROC curve. The ranges for the area under the ROC curve (AUC) values were as follows: 0.9–1.0, excellent; 0.8–0.9, good; 0.7–0.8, moderate; 0.5–0.7, unsatisfactory. The cut‐off point with the highest sensitivity and specificity was chosen.

## 3 Results

### 3.1 Clinical Characteristics of Patients and Comparison Between the Study and Control Groups

The clinical characteristics of patients with EMV treated with UAE in the study and control groups are presented in Table 1. Patients in the study group were younger (*p* = 0.031), had higher parity (*p* = 0.025), and a longer interval since the last pregnancy termination (*p* = 0.003) compared with those in the control group. No significant differences were observed between the two groups regarding gravity, number of cesarean sections, duration of abnormal bleeding, serum hCG positivity, previous mode of prior pregnancy termination, cervical involvement, degree of anemia, or use of compound oral contraceptives (COCs) after UAE (*p* > 0.05).

**TABLE 1:** Clinical characteristic among women with EMV in study and control groups.

	Study group (*N* = 16)	Control group (*N* = 94)	*p*
Preoperative features			
Age, year	28.00 (23.75–34.75)	33.00 (29.00–36.00)	0.031^a^
Gravity, times	3.00 (1.25–4.00)	3.00 (2.00–4.00)	1.000^a^
Parity, times	1.00 (0.25–1.00)	1.00 (1.00–2.00)	0.025^a^
Number of cesarean deliveries, times	1.00 (0.00–1.00)	1.00 (0.00–1.00)	0.521^a^
Interval since the last pregnancy termination, year	0.75 (0.31–5.25)	0.17 (0.08–0.92)	0.003^a^
Duration of abnormal bleeding, day	2.00 (1.00–127.25)	3.50 (1.00–25.00)	0.930^a^
Serum hCG positive (≧ 5.3 IU/L)			
Yes	5 (31.25%)	15 (15.96%)	0.160^b^
No	11 (68.75%)	79 (84.04%)	
Previous mode of prior pregnancy termination			
Cesarean delivery	5 (31.25%)	27 (28.72%)	0.459^c^
Vaginal delivery	2 (12.50%)	5 (5.32%)	
Uterine curettage	7 (43.75%)	55 (58.51%)	
Medical abortion	2 (12.50%)	7 (7.45%)	
Diameter of uterine EMV, cm	4.45 (3.45–7.25)	3.40 (2.70–4.40)	0.009^a^
Involvement of the uterine fundus			
Yes	10 (62.50%)	4 (4.26%)	< 0.001^c^
No	6 (37.50%)	90 (95.74%)	
Involvement of the cervix			
Yes	2 (12.50%)	36 (38.30%)	0.050^c^
No	14 (87.50%)	58 (61.70%)	
Severity of anemia			0.978^c^
No anemia	6 (37.50%)	36 (38.30%)	
Mild anemia	4 (25.00%)	16 (17.02%)	
Moderate anemia	6 (37.50%)	32 (34.04%)	
Postoperative features			
Using COC after UAE			0.423^b^
Yes	6 (62.50%)	26 (27.66%)	
No	10 (37.50%)	68 (72.34%)	
Bleeding time after UAE, day	38.50 (18.50–43.50)	6.00 (3.00–11.00)	< 0.001^a^
EMV mass changes after UAE			< 0.001^c^
Increase	8 (50.00%)	0 (0)	
No significant change	6 (37.50%)	24 (25.53%)	
Decrease	2 (12.50%)	44 (46.81%)	
Disappear	0 (0)	26 (27.66%)	

^a^ Nonparametric test was used.

^b^ Fisher's exact test was used.

^c^ chi‐square test (*χ* ^2^ test) was used.

For the postoperative features, compared with the control group, patients in the study group had a longer duration of bleeding after UAE (*p* < 0.001). Additionally, a greater proportion of patients in the study group exhibited increased EMV mass compared with the control group (*p* < 0.001).

### 3.2 Firth‐Corrected Binary Logistic Regression Analysis of Factors Associated With UAE Treatment Failure

Firth‐corrected binary logistic regression analysis was conducted to examine whether age, parity, interval since the last pregnancy termination, diameter of uterine EMV, involvement of the uterine fundus, and involvement of the cervix were associated with UAE treatment failure in EMV patients (Figure 3). Logistic regression results demonstrated that the diameter of uterine EMV (OR: 3.23, 95% CI: 1.59–8.51, *p* < 0.001) and uterine fundus involvement (OR: 74.03, 95% CI: 7.88–697.40, *p* < 0.001) were significantly associated with UAE treatment failure. As shown in Figure 4, the AUC of EMV diameter was 0.71 (95% CI: 0.58–0.84, *p* = 0.009). When the EMV diameter exceeded 3.85 cm, the sensitivity and specificity were 62.75 and 61.70, respectively.

**FIGURE 3:**
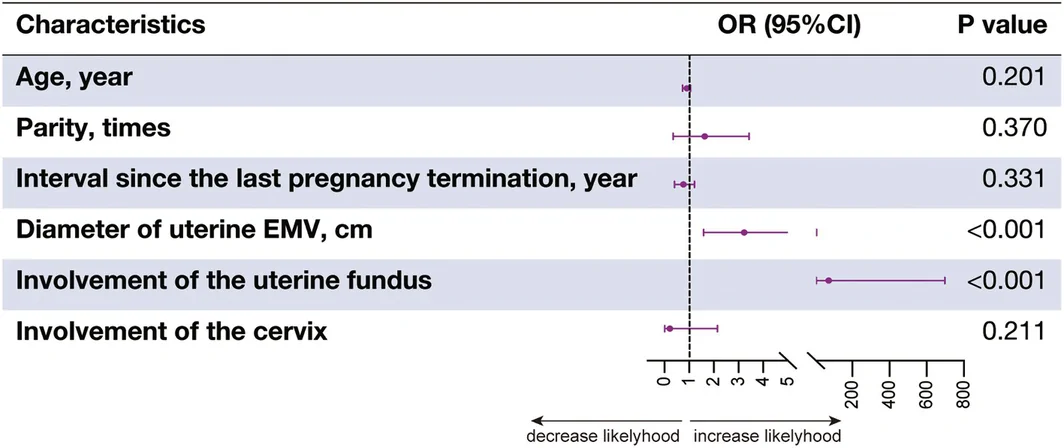
Plot forest chart shows the factors related to failure treatment of EMV.

**FIGURE 4:**
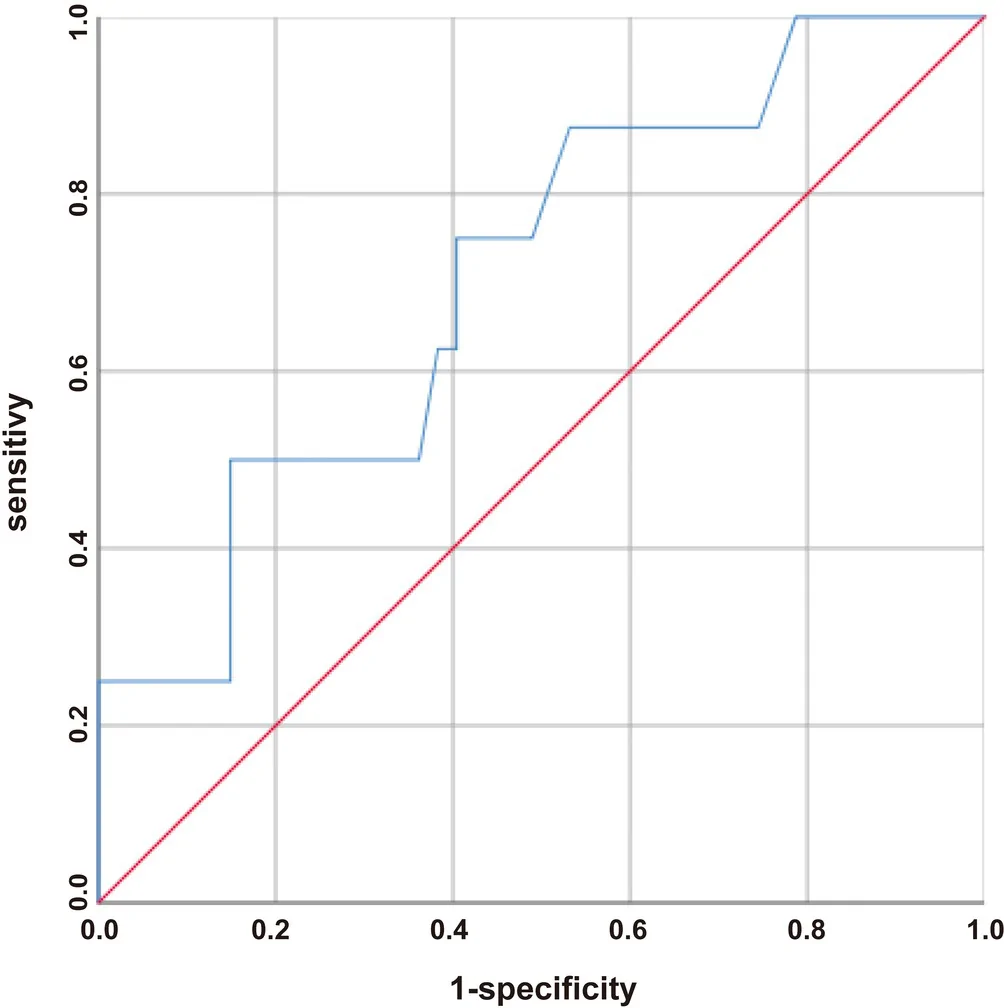
ROC curve of diameter of uterine in predicting failure treatment of EMV.

## 4 Discussion

EMV is characterized by increased vascular proliferation within the myometrium and often results in abnormal uterine bleeding, irrespective of the presence or absence of residual gestational tissue (Van Schoubroeck et al. 2004). This condition can cause significant complications, including physical discomfort and psychological distress, thereby increasing healthcare costs and highlighting the need for effective management strategies. EMV is of particular concern because it may complicate uterine surgery or interventional procedures, especially when not accurately identified by imaging modalities such as ultrasound or MRI. Although UAE has been widely used as a therapeutic approach, its limitations include variable success rates and the risk of treatment failure (Gingold and Bradley 2020; Haldorsen and Salvesen 2012). A more comprehensive understanding of EMV and its clinical implications is therefore essential for improving patient care and treatment outcomes.

Markedly EMV identified on Doppler ultrasound is a common non‐specific imaging manifestation that brings challenges to differentiate physiological or benign hypervascular uterine changes from invasive gestational trophoblastic disease (GTD). Benign myometrial hypervascularity, frequently associated with adenomyosis or intramural ectopic lesions, originates from localized gestational implantation or ectopic endometrial proliferation, presenting as well‐demarcated lesions without destructive myometrial infiltration and accompanied by gradually declining serum β‐hCG levels. In contrast, hypervascular lesions caused by GTD derive from uncontrolled invasive proliferation of cytotrophoblast and syncytiotrophoblast, with persistently elevated or fluctuating β‐hCG. Conventional ultrasound fails to define the depth of interstitial invasion, whereas pelvic MRI enables accurate discrimination. MRI distinctly visualizes irregular, infiltrative interstitial vascular destruction characteristic of trophoblastic lesions, while benign increased myometrial perfusion displays intact surrounding myometrial architecture without invasive destruction. Combined serial β‐hCG monitoring and preoperative MRI effectively avoid unnecessary hysterectomy, and histopathological examination serves as the definitive diagnostic criterion (Khalifa et al. 2025).

Prophylactic endovascular interventions are known to be effective for abnormal uterine bleeding in patients with EMV, placenta accreta spectrum (PAS), and placenta previa. A study comprising 3 randomized controlled trials and 59 observational studies demonstrated that all prophylactic procedures could reduce perioperative blood loss. The complication rate of the UAE group is approximately 1.01%, which is markedly lower than that of resuscitative endovascular balloon occlusion of the aorta group (5.52%) and internal iliac artery balloon occlusion group (1.61%). Compared with patients who did not receive prophylactic endovascular intervention, UAE can significantly reduce intraoperative blood loss (an average reduction of approximately 1.8 L) and the rate of peripartum hysterectomy (with a roughly 50% reduction in risk) (Bonavina et al. 2025). In this study, the effective rate of UAE for the EMV was 85.45%, which confirms that UAE yields satisfactory outcomes in managing abnormal uterine bleeding.

The present study investigated phenotypic factors associated with EMV in patients treated with UAE. Using a retrospective design, we analyzed extensive clinical data spanning a decade to identify predictors of treatment failure and characterize patients with EMV. Our findings suggest that the diameter of the EMV mass and its involvement of the uterine fundus are significant predictors of treatment outcomes. These results emphasize the need to recognize risk factors for treatment failure and highlight the potential of tailoring treatment strategies to enhance clinical outcomes in EMV management (Thomassin‐Naggara et al. 2010).

This study contributes several novel insights to the existing literature on EMV and treatment outcomes following UAE. A key finding is the identification of specific anatomical factors, namely EMV mass diameter and uterine fundal involvement, as significant predictors of treatment failure. This contrasts with previous studies that primarily emphasized demographic characteristics without addressing structural and physiological features influencing treatment efficacy. For example, while the study by Kublickiene et al. (Kublickiene et al. 1997) focused on vascular responses in myometrial arteries, our research extends this knowledge to a clinical context, underscoring the importance of individualized evaluation based on EMV characteristics.

The implications of these findings extend beyond academic interest and may directly influence clinical practice and policy‐making. The demonstrated associations between patient characteristics and treatment outcomes reinforce the importance of personalized approaches in gynecological interventions. By integrating these predictors into clinical decision‐making, healthcare providers can optimize patient management. For instance, younger patients with larger EMV masses or fundal involvement may benefit from closer monitoring or alternative therapeutic strategies. Such tailored management has the potential to improve prognosis while reducing the healthcare burden associated with treatment failure.

However, this study has several limitations. The retrospective design introduces inherent biases, including the possibility of incomplete data and unmeasured confounding variables. Furthermore, the relatively small and homogeneous sample restricts the generalizability of the findings. Future investigations would benefit from a multicenter approach to validate these results across diverse populations and clinical settings. In addition, a longitudinal study design could provide deeper insights into the long‐term outcomes of different treatment strategies, particularly given the dynamic nature of myometrial vascularity over time (Haldorsen and Salvesen 2012; Sipos et al. 2013). Unfortunately, due to the retrospective design of this study, UA parameters, including peak systolic velocity (PSV) and arterial diameter were not collected. Therefore, we failed to explore whether these indicators could affect the treatment success rate. Also, the study findings may not be generalizable to all patients with obstetric bleeding, particularly those with severe conditions (e.g., shock). While the present study offers valuable information for the management of EMV, further research is required to fully clarify the complexity of this condition and its therapeutic options.

In conclusion, this study provides important insights into the clinical characteristics and treatment outcomes of UAE for EMV. The identification of key risk factors and efficacy indicators highlights the significance of personalized patient management in improving treatment success. These results contribute to the current body of evidence and establish a foundation for future research aimed at optimizing therapeutic strategies and enhancing patient care in this complex clinical condition. By addressing these objectives, this study aims to contribute valuable evidence to the field of gynecological healthcare.

## Funding

This work was supported by the Medical Science and Technology Project of Zhejiang Province (No. 2025696389).

## Conflicts of Interest

The authors declare no conflicts of interest.

## Data Availability

The data supporting the findings of this study are available from the corresponding author upon reasonable request. The data are not publicly available due to privacy and ethical restrictions.
